# Supplementary material for: Asynchronous magnetic resonance elastography: Shear wave speed reconstruction using noise correlation of incoherent waves
Source: Magn Reson Med. 2022 Oct 27;89(3):990–1001. doi: 10.1002/mrm.29502 (PMC9792433; doi:10.1002/mrm.29502)
Supplement: Supplementary file 1 — DATA S1 MATLAB code used to generate the numerical simulations of Figure 1 [file MRM-89-990-s002.zip › k-Wave/helpfiles/makeCartCircle.html]

makeCartCircle :: Functions (k-Wave)


# makeCartCircle

Create a 2D Cartesian circle or arc.

## Syntax

```
circle = makeCartCircle(radius, num_points)
circle = makeCartCircle(radius, num_points, center_pos)
circle = makeCartCircle(radius, num_points, center_pos, arc_angle)
circle = makeCartCircle(radius, num_points, center_pos, arc_angle, plot_circle)
```

## Description

`makeCartCircle` creates a 2 x `num_points` array of the Cartesian coordinates of points evenly distributed over a circle or arc (if `arc_angle` is given). The plot that is displayed after calling `makeCartCircle(10, 100, [0,0], (215/180)*pi, true);` is given below.

## Inputs

|  |  |
| --- | --- |
| `radius` | circle radius [m] |
| `num_points` | number of points on the circle |

## Optional Inputs

|  |  |
| --- | --- |
| `center_pos` | [x, y] position of the circle center [m] (default = [0, 0]) |
| `arc_angle` | arc angle for incomplete circle [radians] (default = 2\*pi) |
| `plot_circle` | Boolean controlling whether the Cartesian points are plotted using `plot` (default = false) |

## Outputs

|  |  |
| --- | --- |
| `circle` | 2 x `num_points` array of Cartesian points |

## Examples

- Homogenous Propagation Medium
- Loading External Image Maps
- Heterogeneous Propagation Medium
- Saving Movie Files
- Defining A Gaussian Sensor Frequency Response
- Comparison Of Modelling Functions
- Modelling Sensor Directivity in 2D
- Modelling Sensor Directivity in 3D
- 2D Time Reversal For A Circular Sensor
- Image Reconstruction With Compensation For Acoustic Absorption
- Controlling The Absorbing Boundary Layer
- Optimising k-Wave Performance

## See Also

`cart2grid`, `makeCartSphere`, `makeCircle`
